# Supplementary material for: Cholesterol-binding motifs in STING that control endoplasmic reticulum retention mediate anti-tumoral activity of cholesterol-lowering compounds
Source: Nat Commun. 2024 Mar 29;15:2760. doi: 10.1038/s41467-024-47046-5 (PMC10980718; doi:10.1038/s41467-024-47046-5)
Supplement: Supplementary file 1 — Supplementary Information [file 41467_2024_47046_MOESM1_ESM.pdf]

Supplementary Information for

Cholesterol-binding motifs in STING that control endoplasmic retention mediate anti-tumoral activity of cholesterol-lowering compounds

By Zhang et al.

This file contains

1 Supplementary table

11 Supplementary Figures

Supplementary Table 1. Information on the single guide RNA used for generating Knockout cell lines.

Electroporation delivery	gRNA name	gRNA seq
	AAVS1	GGGGCCACUAGGGACAGGAU
	LSS-gRNA	CUCAGGUGUCUGCGGCGCCG
	ABCG1-gRNA1	AGAGAUGACGGAGCCCAAGU
	ABCG1-gRNA2	CAUCUCUGCAGAGUAACUGC
	CAV1-gRNA1	AUCUCUACACCGUUCCCAUC
	CAV1-gRNA2	GCCCUGUUCCCGGAUGGGAA
	OSBP-gRNA1	GGCGACGGAGCUGAGAGGAG
	OSBP-gRNA2	AUGGCGGCGACGGAGCUGAG
	SIGMAR1-gRNA1	GCCAGCUCGUGGUCCAGCCC
	SIGMAR1-gRNA2	GCCAGGGCUGGACCACGAGC
	SOAT1-gRNA1	UGCUGACUUUUCAAUGAGAU
	SOAT1-gRNA2	AUUGAAGCCAUUUUUUAUGA
	VAPA-gRNA1	UGAAGACUACAGCACCUCGC
	VAPA-gRNA2	CACCUCGCCGGUACUGUGUG
Lentivirus delivery	SOAT1-gRNA5063	GCCCUUCAGCGCUCGUGUUC

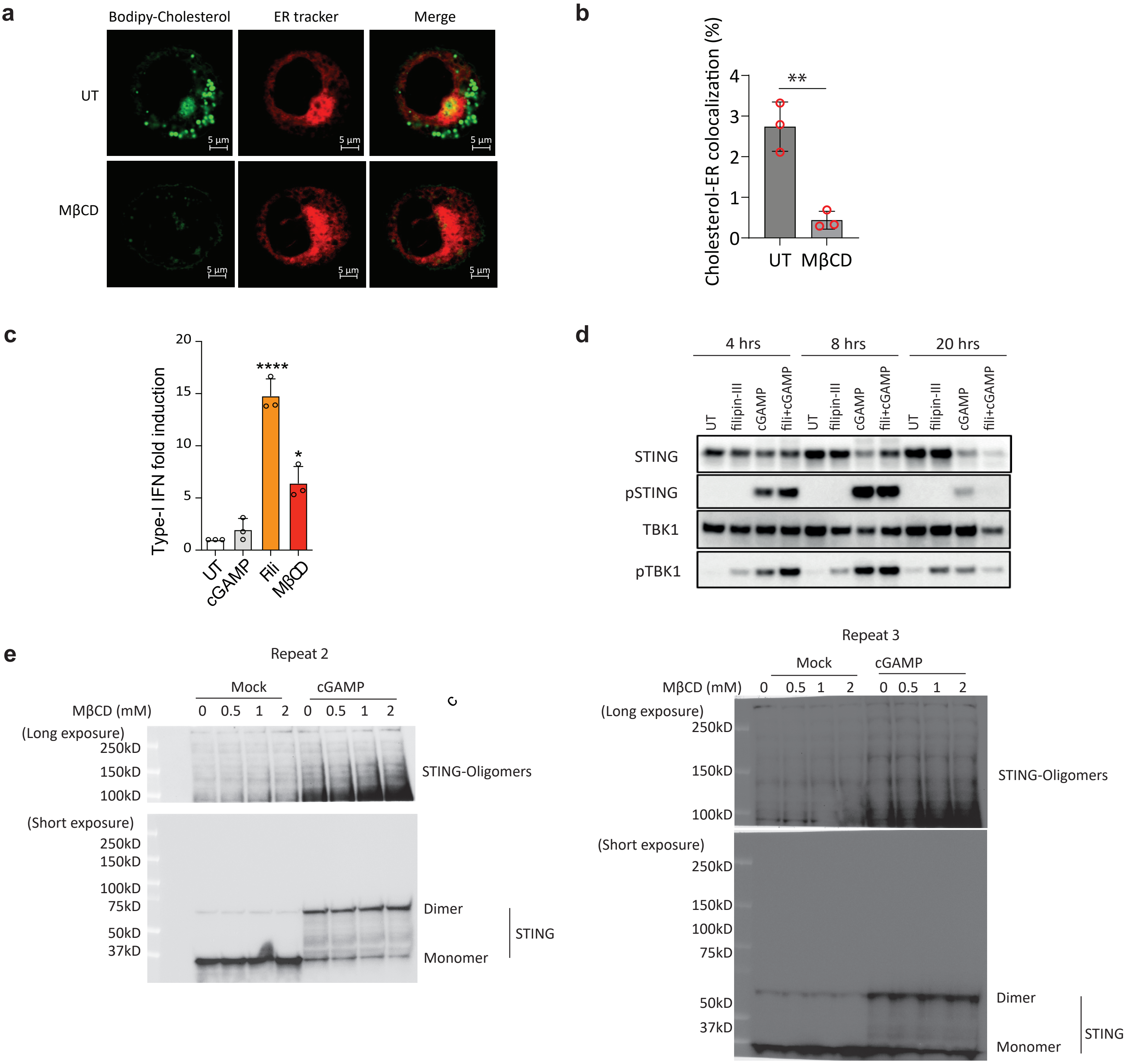

Supplementary Figure 1. Cholesterol depletion increases STING activation. a,b The degree of colocalization of ER and cholesterol in THP1 cells, were done by probing cells with ER-Tracker™ Red and BODIPY-Cholesterol and then treat with MβCD (2mM) for 2 hours. (**a**) Cells were subjected to Zeiss LSM 800 confocal microscope to acquire images. The images were then processed with the Zen Blue software 3.8 (Zeiss). (**b**) The colocalization of ER and cholesterol was analyzed using the ImageStream system, where the level of colocalization was determined using IDEAS 6.3. The quantitative results are presented as the mean value ± SD. Statistical significance was calculated using one-way ANOVA with Tukey's multiple comparison correction. **c** The relative expression of type I IFN secreted in moDCs treated with cGAMP (5 µM), filipin-III (1 µg/ml), or MβCD (1 mM) for 24 hours were analyzed using HEK-blue type I IFN reporter cells. The average of three different donors was used for analysis. Statistical significance was calculated using one-way ANOVA with Tukey's multiple comparison correction (*, $p < 0.05$; ****, $p < 0.0001$). **d** Human moDCs were treated with filipin-III (1 µg/ml), cGAMP (5 µM), or a combination for a total of 4, 8, or 20 hours. The level of STING, Phospho-STING, TBK1, and phospho-TBK1 protein expression was determined by immunoblotting. **e** Immunoblot analysis of STING dimer and oligomer formation in THP-1 cells treated with increasing doses of MβCD before stimulation with cGAMP (50 µg/ml) for 1 hour. The data displayed here are from two out of three independent repeated experiments, while the data from the first experiment are shown in Fig1b.

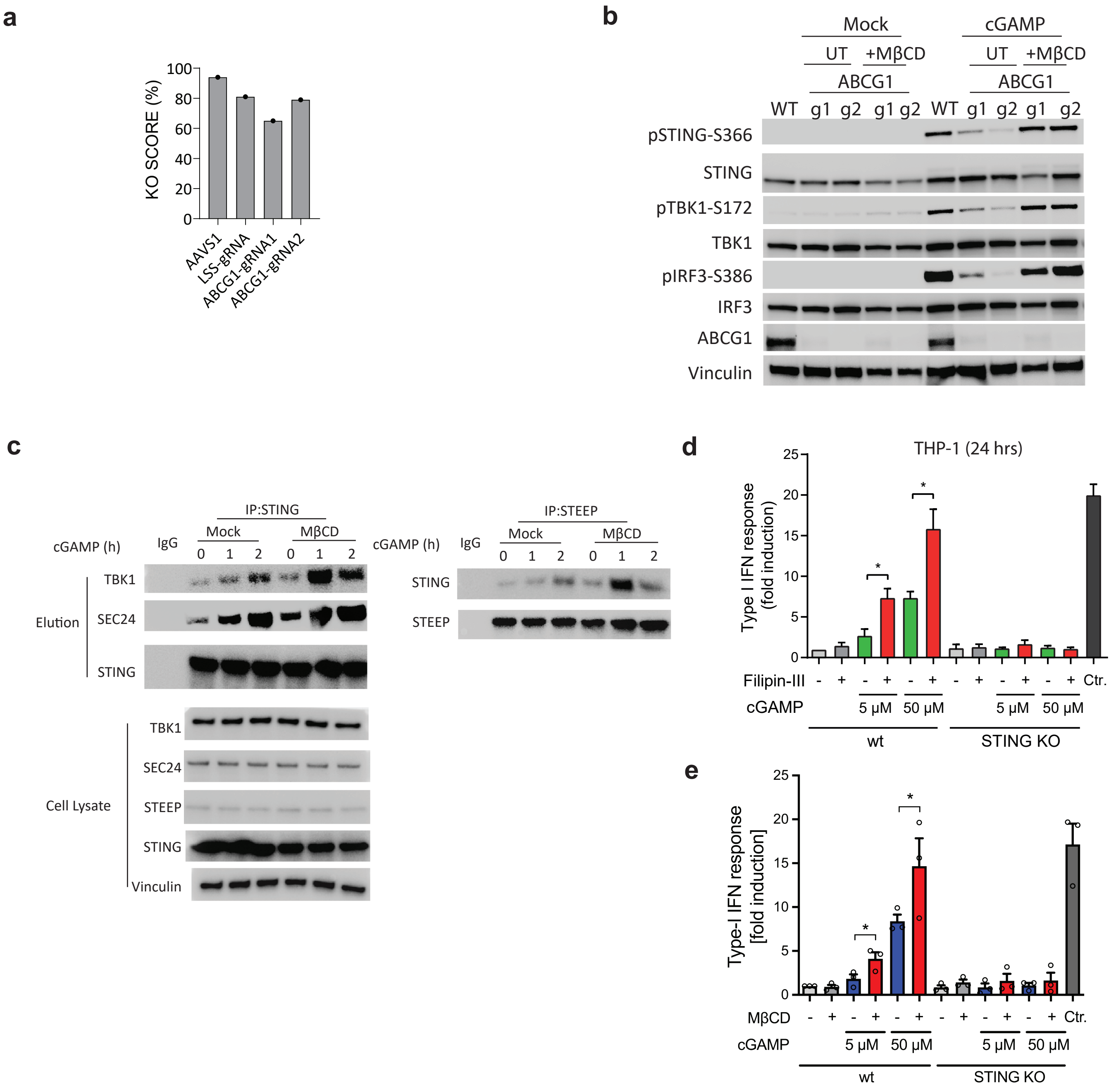

Supplementary Figure 2. Cholesterol depletion increases STING-mediated IFN response. **a**) Efficiency of the indicated sgRNA targeting in THP1 cells shown as KO scores (%) was calculated based on the ICE analysis tool from Synthego. **b** THP1 cells were electroporated with complexes of Cas9 protein and AAVS1/ABCG1 sgRNAs. After 5 days, cells were pre-treated with vehicle or MβCD (2mM) for 2 hrs and then stimulated with vehicle or cGAMP (50ug/ml) for 1 hour. The degree of phosphorylation of STING, TBK, and IRF3 were analyzed by immunoblotting. The gRNA targeting the AAVS1 Safe Harbor Site was used as control. **c** Immunoblot analysis of STING, SeC24 or TBK1 co-immunoprecipitated with either endogenous expressed STING or STEEP in THP1 cells. Prior to the immunoprecipitation cells were pre-treated with mock or MβCD (2 mM) and then stimulated with cGAMP (50 μg/ml). The data displayed here are from second of three independent repeated experiments, while the data from the first experiment are shown in Fig 1d. **d,e** Type I IFN production from WT or STING-KO THP-1-derived macrophages treated with g) filipin-III (1ug/ml) or h) MβCD (1 mM), and then cGAMP (5 or 50 μM) for 24 hours were analyzed using HEK-blue type I IFN reporter cells. Data represent one of two repeated experiments with three biological replicates. Statistical significance was calculated using one-way ANOVA with Tukey's multiple comparison correction (*, $p < 0.05$).

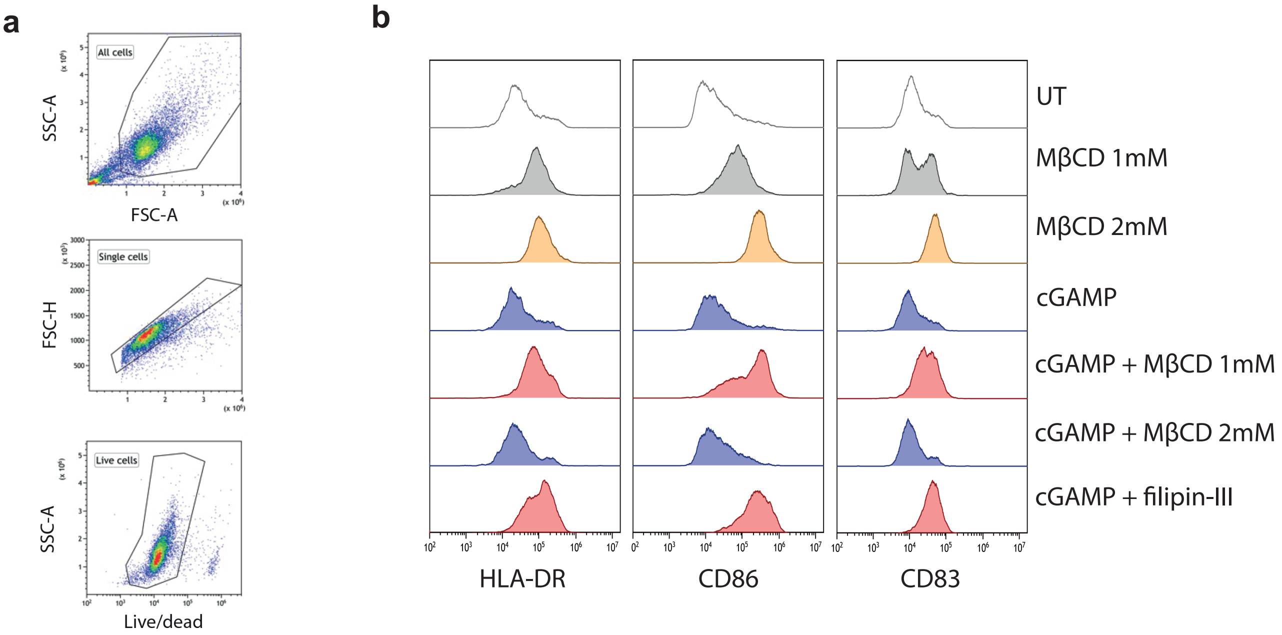

Supplementary Figure 3. Depletion of cholesterol potentiate STING pathway responses. a,b Flow cytometry analysis of the expression of maturation markers HLA-DR, CD86, and CD83 in human moDC treated with cGAMP (5 µM) with or without filipin-III (1 µg/ml) or methyl-β-cyclodextrin (MβCD) (1 or 2 mM). (**a**) Representation of the gating strategy, whereas (**b**) shows the histrogram plots for each marker under each condition. The data are shown as a single replicate from an experimental setup done in tripliates. The combination of multiple samples is shown in Fig 1g.

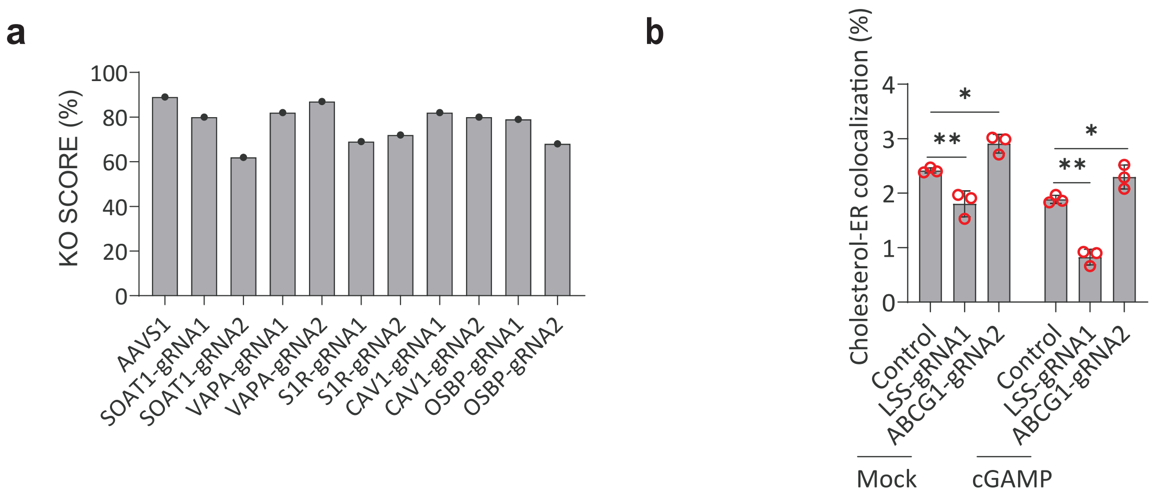

Supplementary Figure 4. a The efficiency of the indicated gRNA targeting in THP1 cells. The KO scores (%) were calculated based on the ICE analysis tool from Synthego. **b** THP-1 cells were electroporated with a complex of Cas9 protein and gRNAs targeted the gene of LSS or ABCG1. Five days post electroporation cells were stimulated with vehicle or cGAMP (50ug/ml) for 1 hour. Cells were probed with ER-Tracker™ Red and BODIPY-Cholesterol and then unstimulated (0 hours) or stimulated with cGAMP (50ug/ml) for 2 hours. The colocalization of ER and cholesterol was analyzed using the ImageStream system, where colocalization was determined using IDEAS 6.2. The gRNA targeting the AAVS1 Safe Harbor Site was used as control. Statistical significance was calculated using one-way ANOVA with Tukey's multiple comparison correction (*, $p < 0.05$; **, $p < 0.01$).

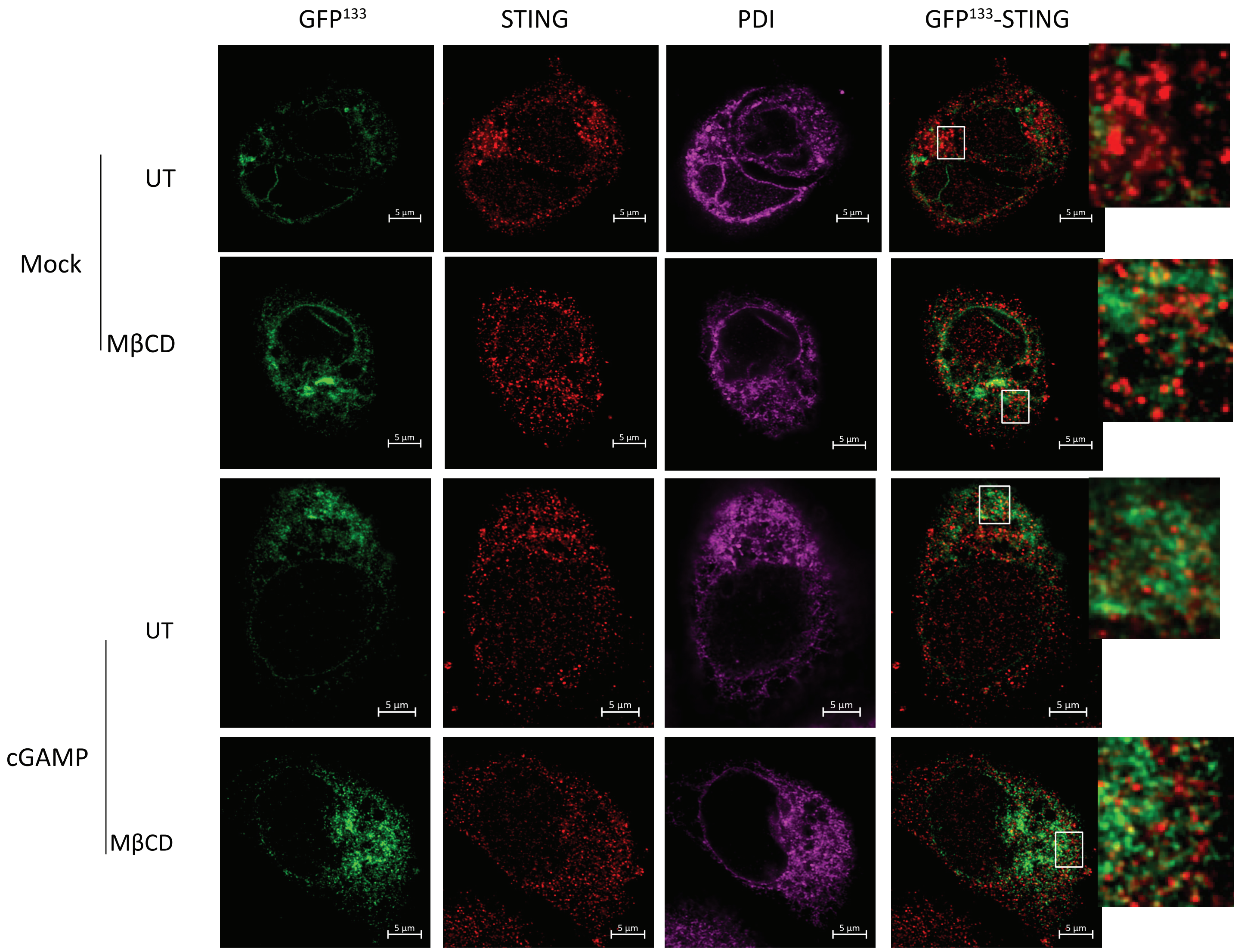

Supplementary Figure 5. The confocal analysis of correlation between ER membrane curvature and STING. A GFP-tagged ALPS (GFP^{133}) construct (ER membrane curvature marker) was transfected into HaCaT cells for 24 h. After 24 hours incubation, cells were pretreated with vehicle or MβCD (2 mM) for 2 h, and then stimulated with vehicle or cGAMP (50ug/ml) for 40 minutes. Cells were fixed using 4% PFA and then probed with mouse anti-PDI (ER marker) and sheep anti-STING. The cells were subjected to Zeiss LSM 800 confocal microscope to acquire images. The images were processed with the Zen Blue software 3.8 (Zeiss).

a

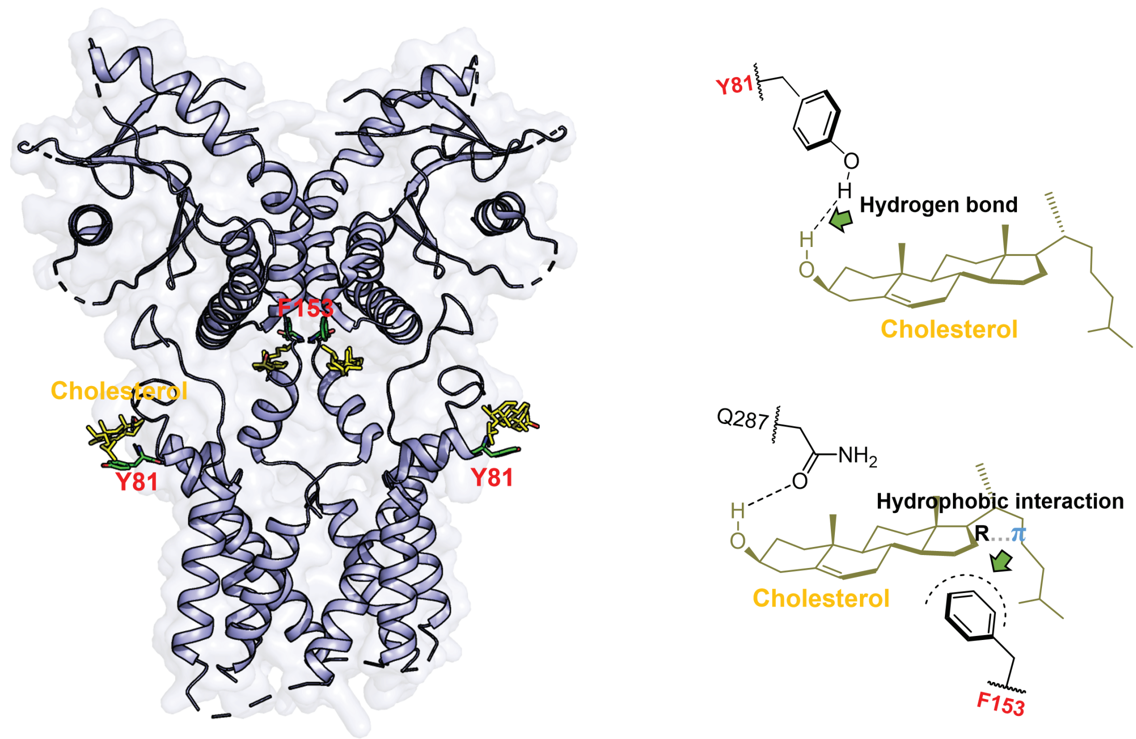

b

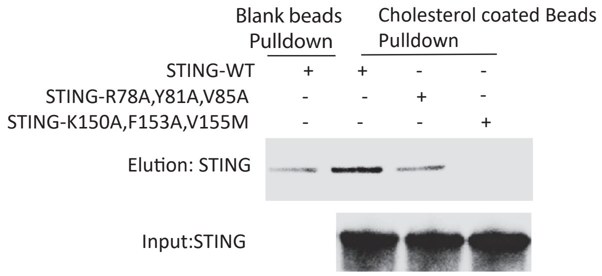

Supplementary Figure 6. a The prediction of cholesterol binding to STING. The left panel shows the cholesterol molecule (yellow) bound to the X-ray structure of STING (gray) (PDB: 6NT6). The right panel shows the potential interaction mode between cholesterol and the two cholesterol binding motifs of STING. **b** The pull-down assay using cholesterol-coated beads.The level of STING protein bound to cholesterol was determined by immunoblotting. (shown as a representative image out of n=3 experiments). The data displayed here are from second of three independent repeated experiments, while the data from the first experiment are shown in Fig4d.

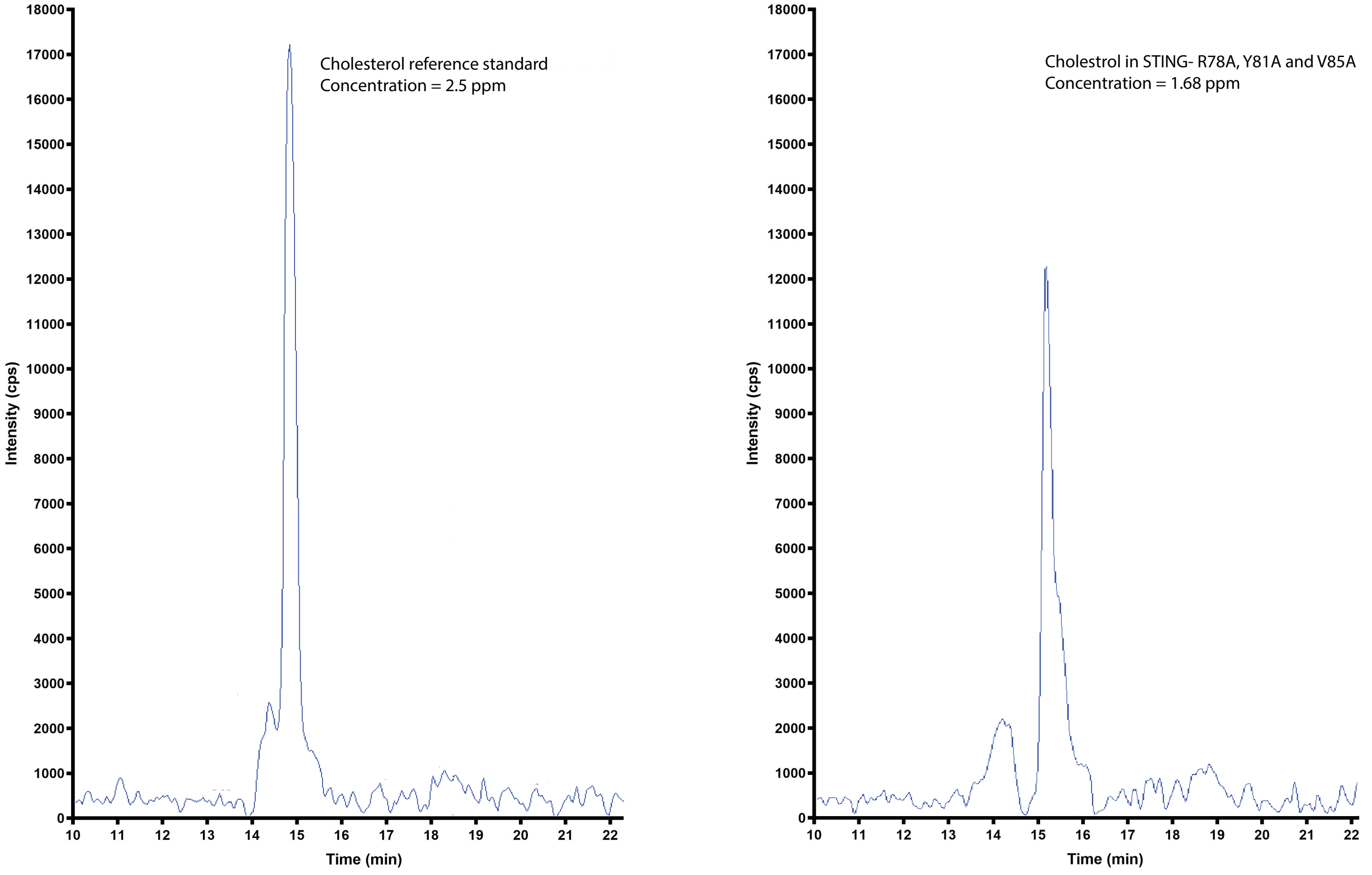

Supplementary Figure 7. Raw chromatogram of the LS/MS evaluation of cholesterol.
Mass spectrometry analysis of cholesterol binding on STING. Immunoprecipitation with FLAG-bead using HEK293T lysates expressing Flag-tagged STING-WT or mutants. Raw dataplot supporting information on figure 4e.

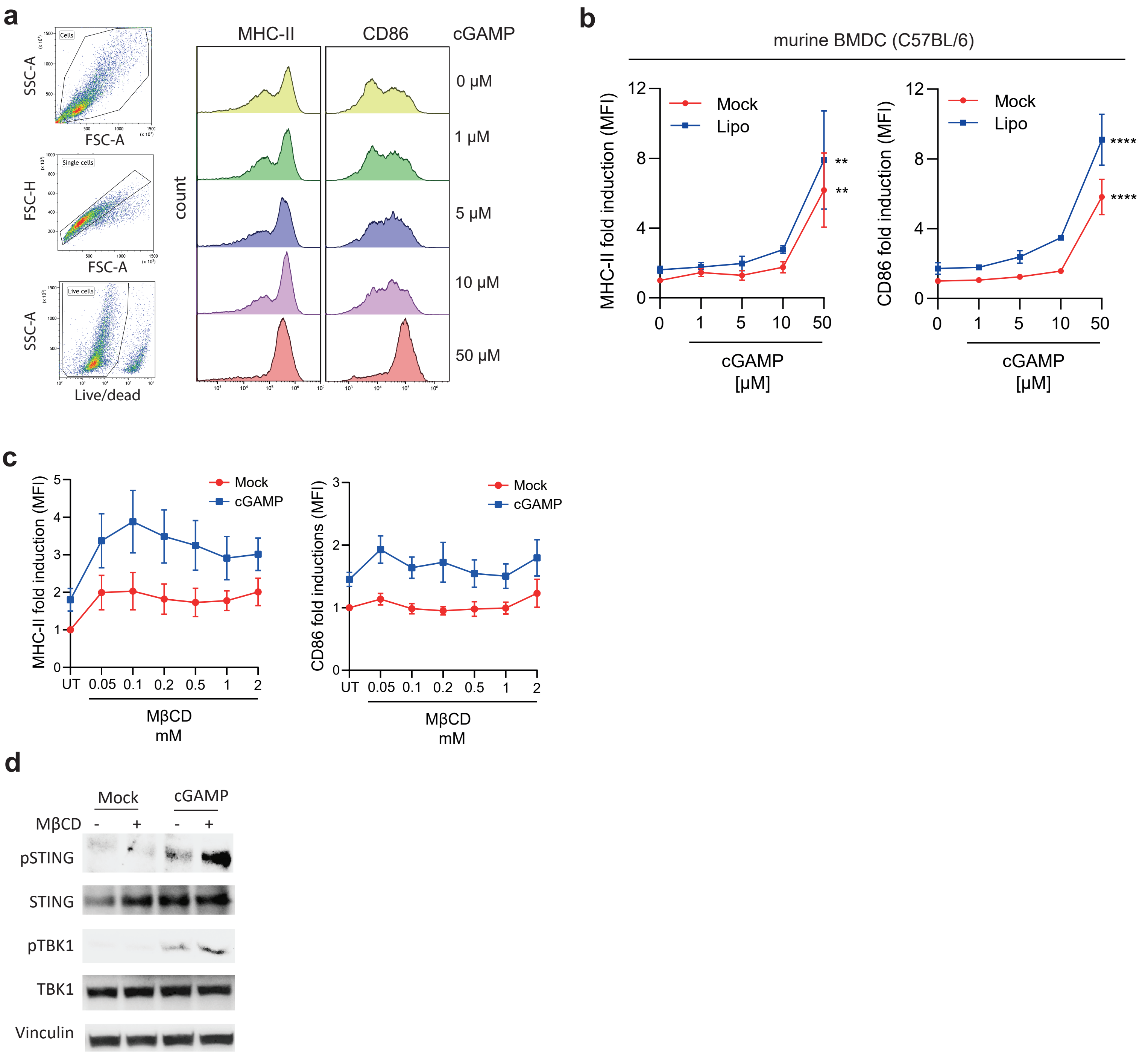

Supplementary Figure 8. Cholesterol depletion augments cGAMP-induced maturation of murine dendritic cells. a,b Expression of maturation markers CD86 and MHC-II in murine bone marrow-derived dendritic cells (BMDCs) (C57BL/6 mice) treated with varying concentrations of cGAMP for 24 hrs. Cells were analyzed using flow cytometry. (**a**) The gating strategy and histograms of one out of three experiments and (**b**) average of all three experiments. The difference from UT was calculated using two-way ANOVA with Tukey's multiple comparison correction (**, $p < 0.01$; **, $p < 0.001$). **c** Expression of maturation markers MHC-II and CD86 in BMDC (C57BL/6) treated with varying concentrations of MβCD and fixed cGAMP concentration (5 µM) for 24 hrs. **d** Immunoblot analysis of whole cell lysates from MC-38 cells treated with 2mM MβCD before stimulation with cGAMP for 1 hour. The indicated proteins were probed using specific antibodies.

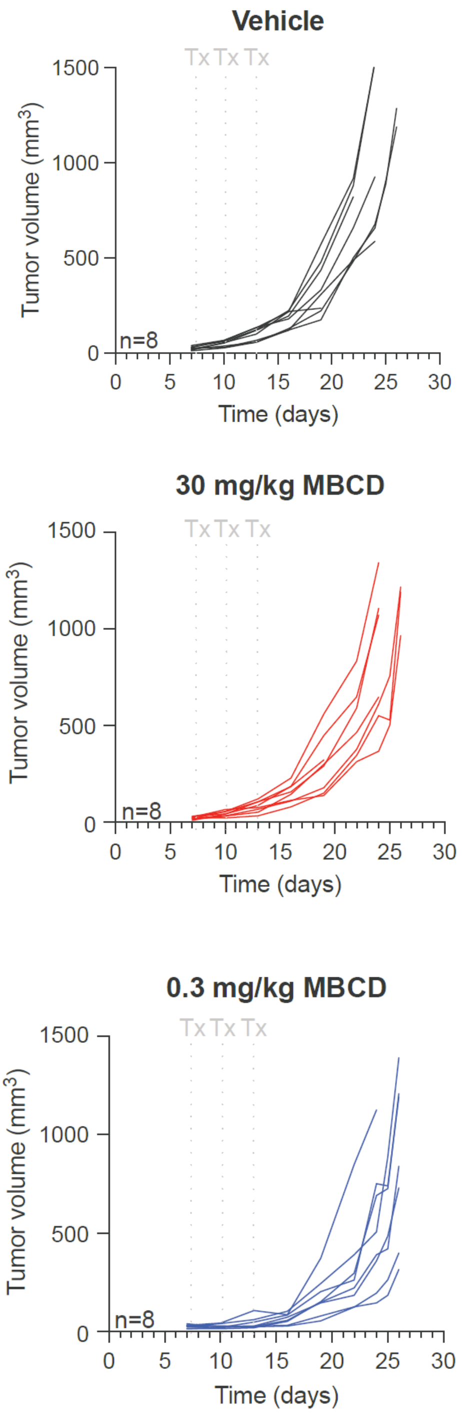

Supplementary Figure 9. Cholesterol depletion through Methyl-β cyclodextrin in vivo stalls tumor growth. C56BL/6 mice (n=8) engrafted with MC38 tumors were either untreated (black lines); treated with high (30 mg/kg, red lines) and low (0.3 mg/kg, blue lines) doses of MβCD by intra tumoral injection on day 7, 10 , and 13. Tumor growth is shown as individual spaghetti plots.

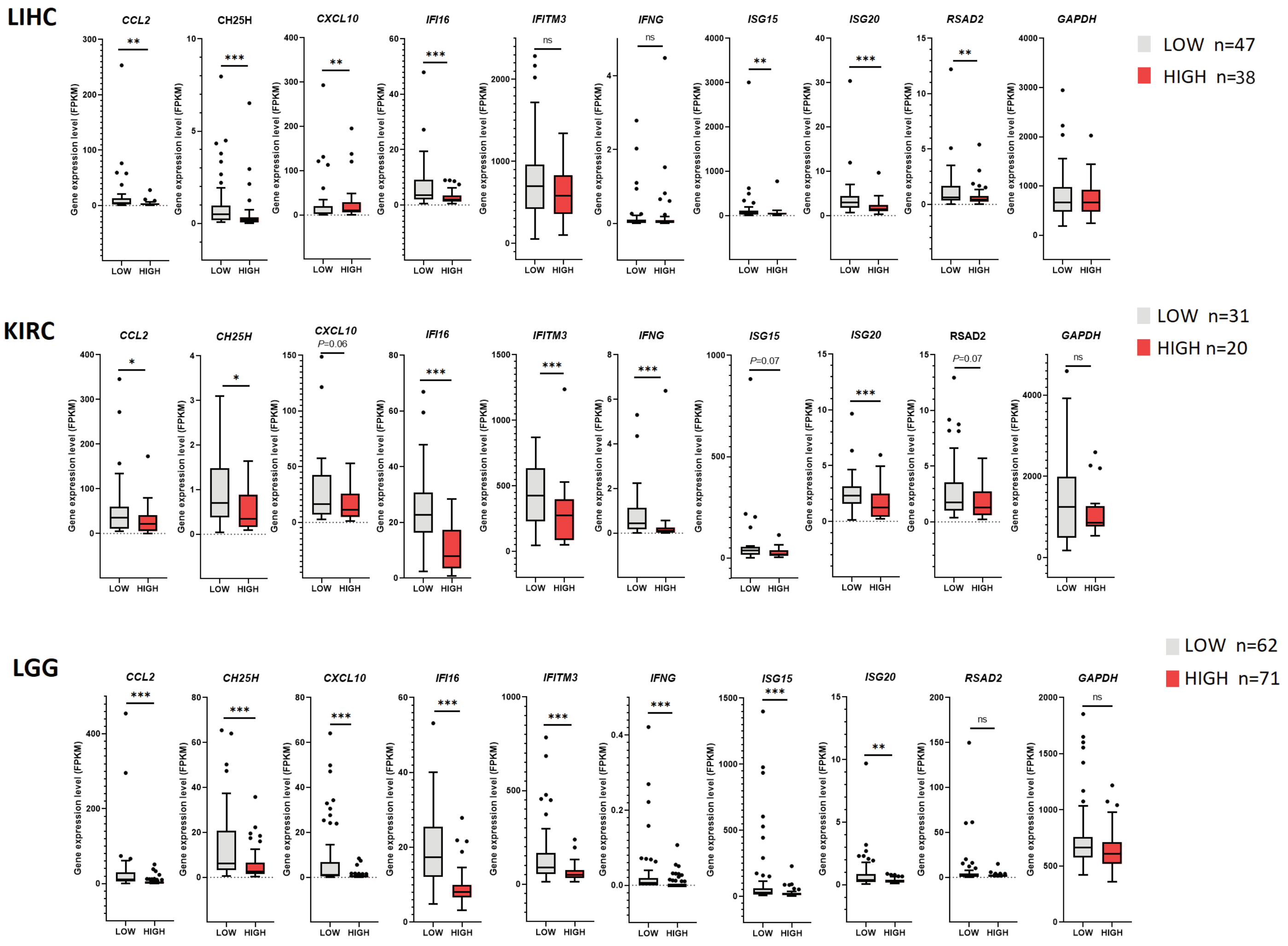

Supplementary Figure 10. Box plots of The Cancer Genome Atlas (TCGA) RNA expression profiles in Liver Hepatocellular Carcinoma (LIHC), Kidney Renal Clear Cell Carcinoma (KIRC), and Low-grade gliomas (LGG). The highest and lowest 25% of cholesterol metabolism were analyzed by comparing cholesterol metabolism-high and cholesterol metabolism-low groups. Statistical analysis was performed using a two-tailed Mann–Whitney test. The upper and lower ends of the boxes represent the upper and lower quartiles, and the horizontal line inside the box is the median of the dataset. The whiskers indicate the upper and lower extremes of the dataset (ns, not significant, *$p < 0.05$, **$p < 0.01$)

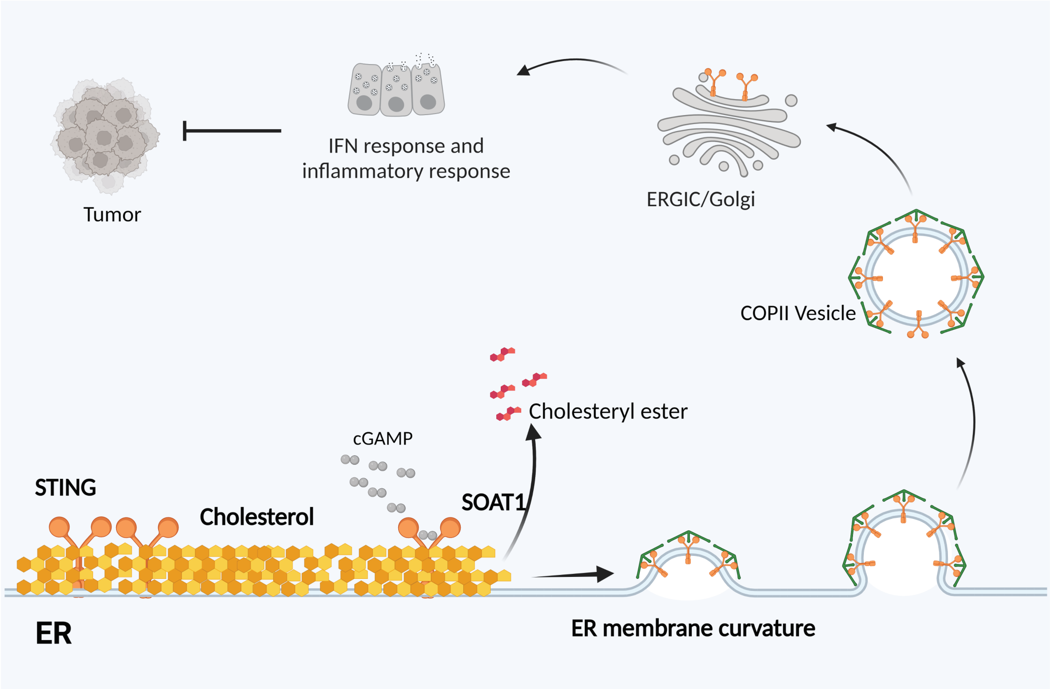

Supplementary Figure 11. Proposed model for action of cholesterol in STING signaling. Cholesterol is shown to retain STING at the endoplasmic reticulum (ER) by inhibiting ER membrane curvature and directly binding to STING. Upon cGAMP stimulation, the enzyme SOAT1 mediates the esterification of cholesterol in the ER, resulting in a reduction of ER cholesterol levels. This reduction creates a window for ER membrane curvature, which allows the COPII complex to assemble and engage STING trafficking from the ER to the Golgi. This process enables IFN and inflammatory responses that are crucial for anti-tumor activities.
