# Supplementary material for: Cholesterol-binding motifs in STING that control endoplasmic reticulum retention mediate anti-tumoral activity of cholesterol-lowering compounds
Source: Nat Commun. 2024 Mar 29;15:2760. doi: 10.1038/s41467-024-47046-5 (PMC10980718; doi:10.1038/s41467-024-47046-5)
Supplement: Supplementary file 3 — Reporting Summary [file 41467_2024_47046_MOESM3_ESM.pdf]

nature portfolio

Corresponding author(s): Professor Martin R Jakobsen, Professor Søren R Paludan, Associate professor Bao-cun Zhang

Last updated by author(s): Feb 23, 2024

Reporting Summary

Nature Portfolio wishes to improve the reproducibility of the work that we publish. This form provides structure for consistency and transparency in reporting. For further information on Nature Portfolio policies, see our Editorial Policies and the Editorial Policy Checklist.

Statistics

For all statistical analyses, confirm that the following items are present in the figure legend, table legend, main text, or Methods section.

n/a	Confirmed	
☐	☒	The exact sample size (*n*) for each experimental group/condition, given as a discrete number and unit of measurement
☐	☒	A statement on whether measurements were taken from distinct samples or whether the same sample was measured repeatedly
☐	☒	The statistical test(s) used AND whether they are one- or two-sided<br>*Only common tests should be described solely by name; describe more complex techniques in the Methods section.*
☐	☒	A description of all covariates tested
☐	☒	A description of any assumptions or corrections, such as tests of normality and adjustment for multiple comparisons
☐	☒	A full description of the statistical parameters including central tendency (e.g. means) or other basic estimates (e.g. regression coefficient) AND variation (e.g. standard deviation) or associated estimates of uncertainty (e.g. confidence intervals)
☐	☒	For null hypothesis testing, the test statistic (e.g. *F*, *t*, *r*) with confidence intervals, effect sizes, degrees of freedom and *P* value noted<br>*Give P values as exact values whenever suitable.*
☒	☐	For Bayesian analysis, information on the choice of priors and Markov chain Monte Carlo settings
☒	☐	For hierarchical and complex designs, identification of the appropriate level for tests and full reporting of outcomes
☐	☒	Estimates of effect sizes (e.g. Cohen's *d*, Pearson's *r*), indicating how they were calculated

Our web collection on statistics for biologists contains articles on many of the points above.

Software and code

Policy information about availability of computer code

Data collection	Images were acquired on Zeiss LSM 800 confocal microscope software ZEN 3.8 and ImageStream MK II Imaging Flow Cytometer the Amnis® IDEAS® 6.2 image analysis software (Amnis, Co., Seattle, WA, USA). Immuno Blots were acquired from ChemiDoc™ Imaging System with Image Lab™ software version 6.0(Bio-Rad). Luciferase activity was measured with Luminoskan Ascent Thermo Scientific SkanIt™ Software (Thermo). qPCR data was acquired on Agilent Technologies AriaMX Real-time PCR System AriaMx PC software v1.0.1408.2642.
Data analysis	Statistical analysis was performed using GraphPad Prism8 to 10. The IDEAS software v6.2 (Amnis Corporation) was used for the colocation analysis of Imagestream images. CRISPR-gRNAs editing frequences of Indel and missense mutations were quantified using the ICE software (http://ice.synthego.com). Confocal Images were analyzed on the Zen 3.8 (Zeiss) and Image J2 software.

For manuscripts utilizing custom algorithms or software that are central to the research but not yet described in published literature, software must be made available to editors and reviewers. We strongly encourage code deposition in a community repository (e.g. GitHub). See the Nature Portfolio guidelines for submitting code & software for further information.

Data

Policy information about availability of data

All manuscripts must include a data availability statement. This statement should provide the following information, where applicable:

- Accession codes, unique identifiers, or web links for publicly available datasets
- A description of any restrictions on data availability
- For clinical datasets or third party data, please ensure that the statement adheres to our policy

All important data generated or analyzed during this study are included in this article. Additional supplementary data are available from the corresponding author upon request

Research involving human participants, their data, or biological material

Policy information about studies with human participants or human data. See also policy information about sex, gender (identity/presentation), and sexual orientation and race, ethnicity and racism.

Reporting on sex and gender	n/a
Reporting on race, ethnicity, or other socially relevant groupings	n/a
Population characteristics	n/a
Recruitment	n/a
Ethics oversight	n/a

Note that full information on the approval of the study protocol must also be provided in the manuscript.

Field-specific reporting

Please select the one below that is the best fit for your research. If you are not sure, read the appropriate sections before making your selection.

☒ Life sciences ☐ Behavioural & social sciences ☐ Ecological, evolutionary & environmental sciences

For a reference copy of the document with all sections, see nature.com/documents/nr-reporting-summary-flat.pdf

Life sciences study design

All studies must disclose on these points even when the disclosure is negative.

Sample size	The number of experiments and replicates was based on standard practices. In general, two or three independent experiments were conducted with minimum three biological replicates. This could ensure we had sufficient statistical power to detect meaningful effects. Additionally, we considered practical constraints and ethical considerations to strike a balance between feasibility and statistical rigor.
Data exclusions	No data were excluded.
Replication	The experiments were replicated or performed independently two or three times to ensure the reliability and reproducibility of our results.
Randomization	For the colocalization images from confocal microscope, Images were taken throughout the slide of each sample using DAPI channel to avoid bias in selection of cells with particular phenotypes. Samples were randomly allocated to each experimental group.
Blinding	Blinding was performed wherever possible: luciferase activity, qPCR, Imagestream and immno blotting were performed on blinded samples which were unblinded after analysis. For experiments not mentioned here, blinding was not possible due to the nature of the experimental design or procedures. For confocal experiments, blinding was not feasible due to the need for direct observation and subjective interpretation of results. Confocal microscopy involves visualizing fluorescently labeled samples under a microscope to capture high-resolution images. The interpretation of these images often requires expertise and subjective judgment to identify specific cellular structures or patterns of fluorescence. Therefore, blinding the experimenters to the treatment conditions would not be practical or meaningful, as they need to directly observe and analyze the images to draw accurate conclusions.

Reporting for specific materials, systems and methods

We require information from authors about some types of materials, experimental systems and methods used in many studies. Here, indicate whether each material, system or method listed is relevant to your study. If you are not sure if a list item applies to your research, read the appropriate section before selecting a response.

Materials & experimental systems

n/a	Involved in the study	
☐	☒	Antibodies
☐	☒	Eukaryotic cell lines
☒	☐	Palaeontology and archaeology
☐	☒	Animals and other organisms
☒	☐	Clinical data
☒	☐	Dual use research of concern
☒	☐	Plants

Methods

n/a	Involved in the study	
☒	☐	ChIP-seq
☐	☒	Flow cytometry
☒	☐	MRI-based neuroimaging

Antibodies

Antibodies used

Antibodies used for Western blotting were rabbit mAb anti-phospho-STING (Ser366) (D7C3S) (1:1000, Cell Signaling Technology), rabbit mAb anti- STING (D2P2F) (1:1000, Cell Signaling Technology), rabbit mAb anti-phospho-TBK1/NAK (Ser172) (D52C2) (1:1000, Cell Signaling Technology), rabbit polyclonal anti-TBK1/NAK (D1B4) (1:1000, Cell Signaling Technology), rabbit anti-ABCG1 (ab52617) (1:1000, abcam), rabbit anti-LSS (1:1000, 13715-1-AP) (Proteintech), rabbit anti-STEEP (1:1000, 24021-1-AP) (Proteintech), rabbit anti-SEC24 (D9M4N) (1:1000, Cell Signaling Technology), rabbit anti-IRF-3 (D83B9) (1:1000, Cell Signaling Technology), rabbit anti-hospho-IRF-3 (Ser386) (E7J8G) (1:1000, Cell Signaling Technology), rabbit anti-ERGIC53/LMAN1 (E2B6H) (1:1000, Cell Signaling Technology), rabbit anti-STING (D1V5L) (1:1000, Cell Signaling Technology), rabbit anti-phospho-STING (Ser365) (D8F4W) (1:1000, Cell Signaling Technology), mouse mAb anti-Vinculin (hVIN1, 1:10000) (Sigma Aldrich). The primary antibody dilution for all experiments, unless otherwise specified, was 1:1000. Secondary antibodies were donkey IgG anti-mouse-HRP (1: 10000, Jackson Immunoresearch) and donkey IgG anti-rabbit-HRP (1:10000, Jackson Immunoresearch). Confocal antibody: sheep anti-STING (1:50 R&D Systems), mouse anti-PDI (1:100, Thermo), rabbit anti-GM130 (1:3000, Cell Signaling Technology),rabbit anti-Sec24 (1:100, D9M4N, CST), secondary antibody Donkey anti-Rabbit/Sheep/Mouse IgG (H+L) Highly Cross-Adsorbed Secondary Antibody, Alexa Fluor™ 488/568/647 (all 1:300, Alexa Fluor; Invitrogen)

Validation

All the above commercial antibodies have been verified by the manufactures. The specific information regarding the species and application can be obtained by referencing the product catalog No and manufacturer's names.

Eukaryotic cell lines

Policy information about cell lines and Sex and Gender in Research

Cell line source(s)

THP1, HaCAT, MC38 and HEK293T cells were obtained from the American Type Culture Collection (ATCC). Human monocyte-derived dendritic cells (moDCs) were generated from whole blood, collected from healthy volunteer donors. Bone marrow-derived dendritic cells were generated from bone marrow isolated from BALB/c or C57BL/6 mice.

Authentication

Cell lines from ATCC were authenticated by the vendor using short tandem repeat (STR) analysis, and were not validated further in our laboratory.

Mycoplasma contamination

All the cells were tested for contamination by mycoplasma, and only cells negative for infection were used in the study.

Commonly misidentified lines
(See ICLAC register)

No commonly misidentified cell lines were used in this study.

Animals and other research organisms

Policy information about studies involving animals; ARRIVE guidelines recommended for reporting animal research, and Sex and Gender in Research

Laboratory animals

C57BL/6 mice

Wild animals

No wild animals were used in this study.

Reporting on sex

female mice were used

Field-collected samples

The study did not involve field-collected samples.

Ethics oversight

The mice were sactifized before any experimental procedures were initiated. Ethical permissions were required and approved according to Danish law, by the regional ethical committee. The animal studies were conducted in accordance with The Animal Ethics Council, license number: 2017-15-0201-01253.

Note that full information on the approval of the study protocol must also be provided in the manuscript.

Plants

Seed stocks	n/a
Novel plant genotypes	n/a
Authentication	n/a

Flow Cytometry

Plots

Confirm that:

☒ The axis labels state the marker and fluorochrome used (e.g. CD4-FITC).

☒ The axis scales are clearly visible. Include numbers along axes only for bottom left plot of group (a 'group' is an analysis of identical markers).

☒ All plots are contour plots with outliers or pseudocolor plots.

☒ A numerical value for number of cells or percentage (with statistics) is provided.

Methodology

Sample preparation	The co-localization of STING-ER and STING-Golgi was determined by the ImageStream MK II Imaging Flow Cytometer (Amnis, Co., Seattle, WA, USA). The cells were fixed using 4% formalin for 20 min at RT, and then pre-permeabilized with 0.2% Triton X 100 for 6 min. The cells were incubated with primary antibodies for 1 h on ice, and then incubated with the Alexa-Fluor-labeled secondary antibodies for 1 h. After every step, the cells were washed with 1xPBS 3 times. Finally, the cells were resuspended in 1xPBS with 2 mM EDTA and 3% BSA.
Instrument	ImageStream MK II Imaging Flow Cytometer
Software	the Amnis® IDEAS® 6.2 image analysis software
Cell population abundance	10,000 single cells with different fluorescence channels were acquired under 60X magnification.
Gating strategy	The cell population gate was performed step by step following the Colocation wizard in the Amnis® IDEAS® 6.2 image analysis software.

☒ Tick this box to confirm that a figure exemplifying the gating strategy is provided in the Supplementary Information.
